# Ixazomib-based induction regimens plus ixazomib maintenance in transplant-ineligible, newly diagnosed multiple myeloma: the phase II, multi-arm, randomized UNITO-EMN10 trial

**DOI:** 10.1038/s41408-021-00590-5

**Published:** 2021-12-07

**Authors:** Roberto Mina, Antonietta Pia Falcone, Sara Bringhen, Anna Marina Liberati, Norbert Pescosta, Maria Teresa Petrucci, Giovannino Ciccone, Andrea Capra, Francesca Patriarca, Delia Rota-Scalabrini, Francesca Bonello, Caterina Musolino, Michele Cea, Renato Zambello, Paola Tacchetti, Angelo Belotti, Claudia Cellini, Laura Paris, Mariella Grasso, Sara Aquino, Lorenzo De Paoli, Giovanni De Sabbata, Stelvio Ballanti, Massimo Offidani, Mario Boccadoro, Federico Monaco, Paolo Corradini, Alessandra Larocca

**Affiliations:** 1 SSD Clinical Trial in Oncoematologia e Mieloma Multiplo, Division of Hematology, University of Torino, Azienda Ospedaliero-Universitaria Città della Salute e della Scienza di Torino, Torino, Italy; 2 Hematology, IRCCS “Casa Sollievo della Sofferenza” Hospital, San Giovanni Rotondo, Italy; 3 Università degli Studi di Perugia - A.O. Santa Maria di Terni, Terni, Italy; 4 Reparto di Ematologia e Centro Trapianto Midollo Osseo, Ospedale Centrale, Bolzano, Italy; 5 Hematology, Department of Translational and Precision Medicine, Azienda Ospedaliera Policlinico Umberto I, Sapienza University of Rome, Rome, Italy; 6 Unit of Clinical Epidemiology, Azienda Ospedaliero-Universitaria Città della Salute e della Scienza di Torino and CPO Piemonte, Torino, Italy; 7 Hematology Division, DAME, University of Udine, Azienda Sanitaria Universitaria Friuli Centrale, Udine, Italy; 8 Multidisciplinary Oncology Outpatient Clinic, Candiolo Cancer Institute, FPO - IRCCS, Torino, Italy; 9 Division of Hematology, University of Messina, Azienda Ospedaliera-Universitaria G. Martino, Messina, Italy; 10 Clinic of Hematology, Department of Internal Medicine (DiMI), University of Genoa and IRCCS Ospedale Policlinico San Martino, Genoa, Italy; 11 Department of Medicine (DIMED), Hematology and Clinical Immunology Section, Padova; University School of Medicine, Padova, Italy; 12 IRCCS Azienda Ospedaliero-Universitaria di Bologna, Istituto di Ematologia “Seràgnoli”, Bologna, Italy; 13 Department of Hematology, ASST Spedali Civili di Brescia, Brescia, Italy; 14 U.O. Ematologia, Ospedale Santa Maria delle Croci, Ravenna, Italy; 15 Division of Hematology, ASST Papa Giovanni XXIII, Bergamo, Italy; 16 Azienda Ospedaliera Santa Croce - Carle, Cuneo, Italy; 17 U.O. Ematologia, IRCCS Ospedale Policlinico San Martino, Genova, Italy; 18 Ematologia, Università del Piemonte Orientale, Novara, Italy; 19 Ematologia, Azienda Sanitaria Universitaria Giuliano Isontina, Trieste, Italy; 20 Divisione di Ematologia con Trapianto, Azienda Ospedaliera Santa Maria della Misericordia di Perugia, Perugia, Italy; 21 Clinica di Ematologia, Azienda Ospedaliero Universitaria Ospedali Riuniti Umberto I-G.M. Lancisi-G. Salesi di Ancona, Ancona, Italy; 22 SC Ematologia, Azienda Ospedaliera SS. Antonio e Biagio e Cesare Arrigo, Alessandria, Italy; 23 Hematology and Bone Marrow Transplant Unit, Fondazione IRCCS Istituto Nazionale dei Tumori di Milano, Milan; University of Milan, Milan, Italy

**Keywords:** Medical research, Haematological diseases, Phase II trials, Myeloma

Bortezomib is a backbone of induction therapies for older patients with multiple myeloma (MM), either in combination with lenalidomide-dexamethasone (VRd) or with daratumumab-melphalan-prednisone (DVMP) [1, 2]. The major limitations to the continuous administration of bortezomib [3] are the risk of developing peripheral neuropathy (PN) [4, 5] and its parenteral administration requiring patient hospitalization. Ixazomib has the advantage of the oral route of administration without the concern for PN, making it a suitable therapeutic option for all-oral combinations and continuous treatment.

Here we present the results of the UNITO-EMN10 trial assessing four ixazomib-based induction regimens followed by ixazomib maintenance in elderly, transplant-ineligible newly diagnosed (ND)MM patients.

Patients with symptomatic NDMM aged ≥65 years or younger but ineligible for autologous stem-cell transplantation (ASCT) could be enrolled. Key inclusion criteria were age ≥18 years; Eastern Cooperative Oncology Group performance status from 0 to 2; and adequate bone marrow, renal, and hepatic reserves (inclusion and exclusion criteria are listed in detail in the Supplementary Appendix). The trial was approved by the institutional review boards or ethics committees at each of the participating centers. All patients gave written informed consent before entering the trial, which was performed in accordance with the Declaration of Helsinki of 1975 (as revised in 2008) and registered on ClinicalTrials.gov as NCT02586038.

This is an open-label, multicenter, multi-arm randomized phase II clinical trial. Patients were randomized to nine 28-day induction cycles of ixazomib (I) 4 mg on days 1, 8, 15 and dexamethasone (d) 40 mg on days 1, 8, 15, 22 or to Id plus either cyclophosphamide (C) 300 mg/m^2^ orally on days 1, 8, 15 or thalidomide (T) 100 mg/day or bendamustine (B) 75 mg/m^2^ iv on days 1, 8, followed by ixazomib maintenance (4 mg on days 1, 8, 15) for up to 2 years.

The trial was designed to select the most promising regimens among the four induction treatments (Id, ICd, IBd, and ITd), conditioning the result on an external target value of 2-year progression-free survival (PFS) of at least 65% to be considered positively for further evaluations, while a 2-year PFS of 50% was considered unsatisfactory. Secondary key endpoints included PFS2 and overall survival (OS) from randomization, PFS from the start of maintenance, response rates (including a minimal residual disease [MRD] detected with a sensitivity of 10^−5^ by flow cytometry in all patients achieving at least a very good partial response [VGPR] at the end of induction), and safety profiles of induction regimens and ixazomib maintenance.

The times of observation were censored on 17 December, 2020. Data were analyzed using R software (version 4.0.2; see the Supplementary Appendix and Tables S1–S2 for the complete statistical details).

A total of 175 patients were enrolled between 1 October 2015 and 5 November 2018 and randomized to Id (42), ICd (61), ITd (61), and IBd (11); of these, 4 did not start treatment (Id, 1; ICd, 2; and ITd, 1) due to consent withdrawal (3) and death (1; Fig. S1). In February 2017, the protocol was amended because of a low enrollment due to the presence—among the oral, ixazomib partners in three of the four study arms—of intravenous bendamustine in the fourth arm (IBd). After enrolling 11 patients in the IBd group, this arm was closed. Furthermore, according to predefined study-stopping rules, after the first 42 patients had been enrolled, the Id arm did not reach the minimum required number of ≥VGPR (4/20 VGPR observed; ≥6/20 required) during the first 4 induction cycles and was therefore closed in March 2018. ICd and ITd arms completed their target enrollment of 61 patients.

The median age of patients enrolled was 74 years (range, 53–88). Patient and disease characteristics are listed in Table S3.

After a median follow-up of 31 months (interquartile range [IQR], 27–37), the median PFS from randomization was 10 months with Id (95% confidence interval [CI] 7–20), 19 with ICd (95% CI 13–27), 12 with Itd (95% CI 10–18), and 14 with IBd (95% CI 3 - not reached [NR]; Fig. 1A). At 2 years, the PFS was 32% in the Id (95% CI 20–50%), 41% in the ICd (95% CI 30–56%), 25% in the ITd (95% CI 16–39%), and 40% in the IBd (95% CI 19–85%) arms. The median PFS2 from randomization was 32 months with Id (95% CI 27-NR), 41 with ICd (95% CI 31-NR), 41 with ITd (95% CI 37-NR), and 41 with IBd (95% CI 21-NR; Fig. 1B). The median OS from randomization was NR in all arms (Fig. 1C).

**Fig. 1:**
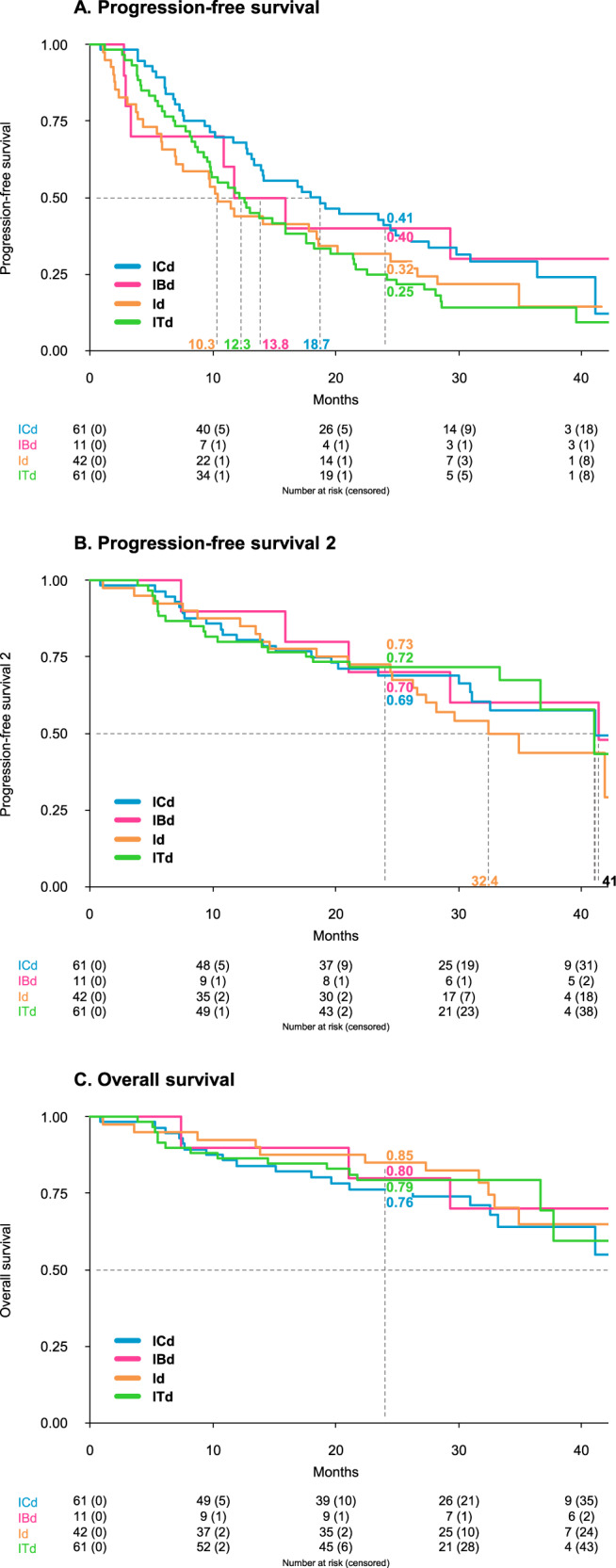
Kaplan‒Meier analyses from randomization by treatment arm. Panel **A** shows Kaplan‒Meier curves for progression-free survival (PFS), Panel **B** for PFS2, and Panel **C** for overall survival (OS) from randomization in patients assigned to ICd (blue line), IBd (magenta line), Id (orange line), and ITd (green line) arms. Id ixazomib-dexamethasone, ICd ixazomib-cyclophosphamide-dexamethasone, ITd ixazomib-thalidomide-dexamethasone, IBd ixazomib-bendamustine-dexamethasone.

The overall response rates (at least a partial response [PR]) after the induction phase were 57%, 75%, 84%, and 73% with Id, ICd, ITd, and IBd, respectively (Table 1). In the intention-to-treat analysis, the rates of MRD negativity at the end of the induction phase were 10%, 3%, 8%, and 9% in the Id, ICd, ITd, and IBd arms, respectively.

**Table 1:** Response rates after induction and maintenance (response-evaluable population).

		All	Id	ICd	ITd	IBd
		*N* = 175	*N* = 42	*N* = 61	*N* = 61	*N* = 11
*Induction*						
ORR		129 (74)	24 (57)	46 (75)	51 (84)	8 (73)
	CR/sCR	14 (8)	4 (10)	6 (10)	3 (5)	1 (9)
	VGPR	56 (32)	6 (14)	22 (36)	26 (43)	2 (18)
	PR	59 (34)	14 (33)	18 (30)	22 (36)	5 (45)
	SD	35 (20)	13 (31)	11 (18)	8 (13)	3 (27)
	PD	3 (2)	2 (5)	-	1 (2)	-
	NE	8 (5)	3 (7)	4 (7)	1 (2)	-
MRD	NEG	12 (7)	4 (10)	2 (3)	5 (8)	1 (9)
*Overall*						
ORR		131 (75)	25 (60)	47 (77)	51 (84)	8 (73)
	CR/sCR	29 (17)	9 (21)	12 (20)	6 (10)	2 (18)
	VGPR	46 (26)	3 (7)	18 (30)	24 (39)	1 (9)
	PR	56 (32)	13 (31)	17 (28)	21 (34)	5 (45)
	SD	33 (19)	12 (29)	10 (16)	8 (13)	3 (27)
	PD	3 (2)	2 (5)	-	1 (2)	-
	NE	8 (5)	3 (7)	4 (7)	1 (2)	-
MRD	NEG	16 (9)	5 (12)	2 (3)	7 (11)	2 (18)

Data are reported as numbers (percentage).

*Id* ixazomib-dexamethasone, *ICd* ixazomib-cyclophosphamide-dexamethasone, *ITd* ixazomib-thalidomide-dexamethasone, *IBd* ixazomib-bendamustine-dexamethasone, *ORR* overall response rate, *CR* complete response, *sCR* stringent CR, *PR* partial response, *VGPR* very good PR, *SD* stable disease, *PD* progressive disease, *NE* not evaluable, *MRD* minimal residual disease, *NEG* negative.

During the induction phase, ixazomib dose reductions were more common in patients receiving triplets (ICd, 24%; ITd, 20%; IBd, 18%) than in those treated with Id (2%). Treatment discontinuation due to adverse events (AEs) occurred more frequently in patients treated with ITd (17%), mostly due to PN (6%), as compared with those who received Id (10%), ICd (12%), or IBd (9%; Fig. S1).

Grade ≥3 hematologic AEs were infrequent (Id, 5%; ICd, 12%; ITd, 8%; and IBd, 18%). At least 1 grade ≥3 non-hematologic AE was reported in 17%, 19%, 48%, and 36% of patients treated with Id, ICd, ITd, and IBd, respectively, with grade ≥3 neurological and dermatologic AEs occurring more frequently in the ITd arm (17% and 13%) than in the Id (7% and 2%), ICd (7% and 2%), and IBd (9% and 0%) arms (Table S4).

Overall, 58% of enrolled patients started ixazomib maintenance (Id, 50%; ICd, 62%; ITd, 62%; and IBd, 45%). After a median follow-up of 25 months (IQR, 21–30) from the start of maintenance, the median PFS was 14.9 months (95% CI 10–19; Fig. S2).

During ixazomib maintenance, 19% of patients improved their response by at least one IMWG category (Fig. S3).

Fifteen % of patients required at least one ixazomib dose reduction. The rate of grade ≥3 hematologic and non-hematologic AEs was low (3 and 14%, respectively). Grade 1–2 PN was observed in 16% of patients, without grade ≥3 events (Table S5).

The primary objective of the trial was the selection of the most promising regimen worth further investigation, provided that a 2-year PFS of at least 65% would have been considered satisfactory. Unfortunately, with a 2-year PFS of 32% with Id, 41% with ICd, 25% with ITd, and 40% with IBd, none of the tested combinations reached the primary endpoint. The 65% target for the primary endpoint had been chosen based on the available data from the VISTA trial at the time of the study design (median time to progression, 24 months) [6, 7], expecting a PFS improvement incorporating ixazomib maintenance after the induction phase. This target may have been over-estimated, considering that the estimated percentage of patients alive and free from progression at 2 years was ~30% with VMP and ~60% with DVMP in the ALCYONE trial [2, 5] and around 40–50% with Rd [8]. Unfortunately, the lack of a control arm including a non-ixazomib-based combination does not allow to draw definitive conclusions.

Acknowledging the limitations of cross-trial comparisons, and also considering ixazomib maintenance in our trial, the combination of ixazomib with an alkylator and corticosteroids resulted in similar overall response rates (ORR, 75% vs. 74%) and median PFS (19 vs. 19 months) as compared with VMP (once-weekly, subcutaneous bortezomib) in the ALCYONE trial, although the rate of complete response obtained with ICd was lower (8%) than that reported with VMP (25%) [5].

Regarding the use of ixazomib maintenance, our results are in line with those reported in the TOURMALINE-MM4 trial, with similar median PFS from the start of maintenance (14.9 vs. 17.4 months) [9] and good tolerability, with no grade 3–4 PN events and with a rate of grade 3–4 infections (2%) lower than that associated with maintenance with continuous daratumumab (11%) [2] or lenalidomide (upper respiratory tract infections, 8%) [10].

Altogether, these results suggest that, due to its tolerability, and particularly due to the lower rate of grade 1–2 (20% vs. 34%) and grade 3–4 (2% vs. 4%) PN as compared to bortezomib, ixazomib may be more suitable as a maintenance therapy in patients in whom a deep cytoreduction has been achieved with more effective induction treatments. Moreover, it may represent an alternative to bortezomib in patients with preexisting PN or when an all-oral regimen is preferable to avoid frequent hospitalization.

Limitations of this study are the lack of a control arm that prevented the possibility to select the best performing regimen through a direct comparison with a standard treatment and the lack of a formal comparison between the investigated arms.

With these caveats, the observed results suggested that the doublet Id was associated with lower ORR (57% vs. 75%) and shorter PFS (median, 10 vs. 19 months) as compared to the triplet ICd. Furthermore, ICd was associated with similar ORR (75% vs. 84%) and ≥VGPR rates (46% vs. 48%) as compared to ITd, but with a longer median PFS (19 vs. 12 months), possibly due to a significantly lower rate of non-hematologic AEs (PN, 7% vs. 2%; dermatologic, 13% vs. 2%) and treatment discontinuation due to AEs (12% vs. 17%), as compared to ITd.

In conclusion, none of the ixazomib-based regimens tested met the primary endpoint of the trial. Among those tested, ICd may represent a viable, all-oral combination for future trials in a subset of older patients. Finally, ixazomib maintenance confirmed to be a well-tolerated approach in elderly MM patients.

## Supplementary information

Supplementary appendix

## Data Availability

After the publication of this article, data collected for this analysis and related documents (including the trial protocol) will be made available to others upon reasonably justified request, which needs to be written and addressed to the attention of the corresponding author Dr. Roberto Mina at the following e-mail address: roberto.mina@unito.it. The sponsor of the trial, the University of Torino (Italy), via the corresponding author Dr. Roberto Mina, is responsible to evaluate and eventually accept or refuse every request to disclose data and their related documents, in compliance with the ethical approval conditions, in compliance with applicable laws and regulations, and in conformance with the agreements in place with the involved subjects, the participating institutions, and all the other parties directly or indirectly involved in the participation, conduct, development, management, and evaluation of this analysis.
