# Supplementary material for: Ixazomib-based induction regimens plus ixazomib maintenance in transplant-ineligible, newly diagnosed multiple myeloma: the phase II, multi-arm, randomized UNITO-EMN10 trial
Source: Blood Cancer J. 2021 Dec 7;11(12):197. doi: 10.1038/s41408-021-00590-5 (PMC8651653; doi:10.1038/s41408-021-00590-5)
Supplement: Supplementary file 1 — Supplementary appendix [file 41408_2021_590_MOESM1_ESM.pdf]

Ixazomib-based induction regimens plus ixazomib maintenance in transplant-ineligible, newly diagnosed multiple myeloma: the phase II, multi-arm, randomized UNITO-EMN10 trial

Supplementary appendix

Supplementary methods **2**

Key inclusion criteria 2

Study design and intervention 2

Statistical analysis 2

Safety *3*

Efficacy *4*

Endpoint assessments 4

Supplementary tables **5**

Table S1. Stopping boundaries for the achievement of a very good partial response (VGPR) 5

Table S2. Stopping boundaries for toxicity 5

Table S3. Patient demographics and disease characteristics at baseline 6

Table S4. Grade 1-2 and grade 3-4 adverse events occurred during the induction phase 7

Table S5. Grade 1-2 and grade 3-4 adverse events occurred during the ixazomib maintenance phase 7

Supplementary figures **8**

Figure S1. Study flow 8

Figure S2. Kaplan–Meier curve for progression-free survival (PFS) from the start of maintenance in patients (n=102) who completed the induction phase and received ixazomib maintenance 9

Figure S3. Response improvement from the induction to the maintenance phase 9

References **10**

Supplementary methods

Key inclusion criteria

Key inclusion criteria were age ≥18 years; Eastern Cooperative Oncology Group performance status of 0 to 2; adequate bone marrow reserves. (platelet count ≥75 x 10^9/L, hemoglobin ≥8 g/dL, absolute neutrophil count (ANC) ≥1.0 x10^9/L); serum creatinine clearance ≥30 mL per minute; adequate hepatic function (alanine aminotransferase up to 3 times and bilirubin up to 1.5 times of the upper normal limit). Exclusion criteria included: peripheral neuropathy grade 2 or higher, uncontrolled active infection and active hepatitis B or C and HIV infection and evidence of current uncontrolled cardiovascular conditions, including uncontrolled hypertension, uncontrolled cardiac arrhythmias, symptomatic congestive heart failure, unstable angina, or myocardial infarction within the past 6 months.

Study design and intervention

The UNITO-EMN10 (NCT02586038) is an open label, multi-arm randomized phase II clinical trial conducted at 21 Italian centers. The primary endpoint was PFS. The aim of the trial was the selection of the most promising regimen with the best 2-year PFS from randomization among four induction combinations: ixazomib-dexamethasone (Id), ixazomib-cyclophosphamide-dexamethasone (ICd), ixazomib-thalidomide-dexamethasone (ITd) and ixazomib-bendamustine-dexamethasone (IBd), all followed by ixazomib maintenance.

Secondary key endpoints included PFS2 and OS from randomization, PFS from start of maintenance, response rates and safety profile of induction regimens and ixazomib maintenance.

Patients were randomized to receive nine 28-day induction cycles of ixazomib 4 mg on days 1, 8,15 and dexamethasone 40 mg on days 1, 8, 15, 22 or Id plus either cyclophosphamide 300 mg/m^2 orally on days 1,8,15 or thalidomide 100 mg/day or bendamustine 75 mg/m^2 iv on days 1, 8, followed by ixazomib maintenance (4 mg on days 1, 8, 15) for up to 2 years.

Statistical analysis

The trial was designed to select the most promising regimen among four induction treatments, followed by maintenance with ixazomib, conditioning the result on an external target value of 2-year PFS of at least 65% to be considered positively for further evaluations, while a 2-year PFS of 50% was considered unsatisfactory. According to the design proposed by Simon, Wittes, and Ellenberg, with an alpha error (one-sided) of 0.05 and a beta error of 0.20, assuming 18 months of accrual and 24 months of minimum follow-up, a total sample size of 244 patients (61 per arm) was required. A continuous monitoring of both efficacy (proportion of patients achieving at least a very good partial response [VGPR] after the first four cycles of treatment) and toxicity (proportion of patients experiencing a toxicity during the first four cycles) was established by defining dual stopping boundaries according to the Bayesian method for simultaneous monitoring of response and toxicity proposed by Thall et al,[1–3] in order to ensure a close monitoring of the trial and a timely termination of the arms with early data suggesting either too low activity or too high toxicity.

The safety endpoint used for monitoring was the proportion of patients experiencing a toxicity (which is defined in the Safety section below) during the first 4 cycles. The efficacy endpoint used for monitoring each arm of the trial was the proportion of patients achieving at least a very good partial response (≥VGPR) after the first 4 cycles of treatment.

The safety and efficacy endpoints were timely recorded, and they analyzed every cohort of 5 patients in each arm.

Historical data on similar patients showed, on average, a VGPR proportion of 45% and a toxicity rate of 30%. Independence was assumed between VGPR and toxicity. The probabilities of VGPR and toxicity for the historical data were modeled by beta distributions [Beta(45, 55) and Beta(30, 70), respectively]. The prior probabilities of VGPR and toxicity for all the experimental regimens were also modeled by beta distributions [Beta(0.9, 1.1) and Beta(0.6, 1.4), respectively], which have the same means as the corresponding beta distributions for the historical data and an Effective Sample Size of 2.

With maximum sample size of 61 patients in each arm, the trial was monitored according to the following stopping boundaries for VGPR listed in Table S1. Furthermore, patients were monitored according to the following stopping boundaries for toxicity listed in Table S2.

Safety

Toxicity was evaluated according to the Common Terminology Criteria for Adverse Events of the National Cancer Institute, version 4.03.[4] Patients were monitored for toxicity to accommodate their tolerance to treatment. According to the statistical design, toxicity of each treatment arm was evaluated at the end of cycle 4. A toxicity rate of ≤30% was considered acceptable. Otherwise, the treatment arm needed to be stopped for excessive toxicity.

Toxicity was defined as the presence of any of the following adverse events (AEs), which were considered by the investigators to be even possibly related to the combination of Id with thalidomide or cyclophosphamide or bendamustine:

1) grade 4 neutropenia (absolute neutrophil count<500/mm^3) lasting at least 7 consecutive days;

2) grade 3 neutropenia with fever and/or infection, where fever was defined as a temperature >38.3 °C;

3) grade 4 thrombocytopenia (platelets < 25,000/mm^3) lasting at least 7 consecutive days;

4) grade 3 thrombocytopenia with clinically significant bleeding, where clinically significant bleeding was defined as a blood loss of >100 cc or the requirement for a red blood cell transfusion;

5) a platelet count <10,000/mm^3;

6) grade 2 peripheral neuropathy with pain or grade ≥3 peripheral neuropathy;

7) grade ≥3 nausea and/or emesis, despite the use of optimal antiemetic prophylaxis, which was defined as an antiemetic regimen including a 5-HT3 antagonist, given in standard doses and according to standard schedules;

8) grade ≥3 diarrhea occurring despite maximal supportive therapy;

9) any other grade ≥3 non-hematologic toxicity, with the exception of grade 3 arthralgia/myalgia and of brief (<1 week) grade 3 fatigue;

10) a delay of more than 2 weeks in the subsequent cycle of treatment due to a lack of adequate recovery from concomitant study drug-related hematologic and/or non-hematologic toxicities;

11) other concomitant study drug-related, grade ≥2, non-hematologic toxicities that, in the opinion of the investigator, required the discontinuation of therapy with the study-drug combinations.

Efficacy

Responses were evaluated after each cycle. Efficacy was measured as the VGPR rate in each treatment arm. The VGPR rate was evaluated after cycle 4 because the literature data showed that the median time to achieve the best response was 4 months of treatment. According to the statistical design, the VGPR rate at cycle 4 was considered adequate if ≥45%. Otherwise, the treatment arm needed to be interrupted. Then, the monitoring of treatment efficacy was performed throughout the induction phase and the maintenance therapy, in order to ensure a sufficient response rate.

Patients were analyzed on an intention-to-treat basis for time-to-event endpoints and response rates; safety was analyzed in patients receiving at least one dose of study drugs. Kaplan–Meier curves were generated for all time-to-event endpoints and log-rank tests were used to compare treatment arms. Response rates and AE rates were compared using the Fisher's exact test. Times of observation were censored on December 17, 2020. Data were analyzed using R software (version 4.0.2).

Endpoint assessments

PFS was calculated from the time of randomization to the date of progression or death or the date the patient was last known to be in remission. PFS2 was calculated from the time of randomization to the date of second relapse/progression or death. OS was calculated from the time of randomization until the date of death for any cause. Patients who did not experience any event were censored at the date of last follow-up. Evaluation of response to the treatment was performed according to the International Myeloma Working Group consensus criteria for response and minimal residual disease assessment in multiple myeloma.[5] Minimal residual disease was assessed in all patients achieving at least VGPR at the end of induction by flow-cytometry (sensitivity of 10^{-5}).

AEs were graded according to the Common Terminology Criteria for Adverse Events of the National Cancer Institute, version 4.03.[4]

Supplementary tables

Table S1. Stopping boundaries for the achievement of a very good partial response (VGPR)

Number of patients in complete cohorts of 5 (inclusive)	Total number of VGPRs to stop the trial (inclusive)
5	0
10	0-1
15	0-3
20	0-5
25	0-6
30	0-8
35	0-10
40	0-11
45	0-13
50	0-15
55	0-17
>60	Always stop

Table S2. Stopping boundaries for toxicity

Number of patients in complete cohorts of 5 (inclusive)	Total number of toxicities to stop the trial (inclusive)
5	4-5
10	6-10
15	9-15
20	11-20
25	13-25
30	15-30
35	17-35
40	19-40
45	21-45
50	22-50
55	24-55
>60	Always stop

Table S3. Patient demographics and disease characteristics at baseline

	All N=175	Id N=42	ICd N=61	ITd N=61	IBd N=11
**Age**					
Median (IQR)	74 (70-77)	73.5 (70-77)	76 (71-78)	73 (70-77)	73 (68-74.5)
>75	70 (40)	16 (38)	32 (52)	20 (33)	2 (18)
**Sex**					
Female	90 (51)	23 (55)	27 (44)	36 (59)	4 (36)
Male	85 (49)	19 (45)	34 (56)	25 (41)	7 (64)
**Type of immunoglobulin**					
BJ	24 (14)	5 (12)	10 (16)	9 (15)	
IgA	28 (16)	11 (26)	7 (11)	7 (11)	3 (27)
IgD	1 (1)	1 (2)			
IgG	110 (63)	21 (50)	38 (62)	43 (70)	8 (73)
Ns	12 (7)	4 (10)	6 (10)	2 (3)	
**Light chain**					
kappa	112 (64)	29 (69)	40 (66)	39 (64)	4 (36)
lambda	63 (36)	13 (31)	21 (34)	22 (36)	7 (64)
**Creatinine clearance**					
30-60	73 (42)	18 (43)	25 (41)	26 (43)	4 (36)
60-90	71 (41)	18 (43)	24 (39)	24 (39)	5 (45)
90+	31 (18)	6 (14)	12 (20)	11 (18)	2 (18)
**ISS**					
I	51 (29)	11 (26)	19 (31)	20 (33)	1 (9)
II	78 (45)	16 (38)	28 (46)	26 (43)	8 (73)
III	46 (26)	15 (36)	14 (23)	15 (25)	2 (18)
**LDH**					
>ULN	18 (10)	3 (7)	6 (10)	9 (15)	
Missing	3	0	1	1	1
**FISH:** t(4;14) t(14;16), del(17p)					
High	35 (23)	5 (13)	15 (28)	12 (23)	3 (33)
Missing	22	4	8	8	2
**R-ISS**					
I	31 (19)	10 (25)	12 (22)	9 (16)	
II	108 (68)	25 (62)	36 (67)	38 (69)	9 (90)
III	20 (13)	5 (12)	6 (11)	8 (15)	1 (10)
Missing	16	2	7	6	1
**Frailty score**					
Fit	77 (44)	19 (45)	21 (34)	28 (46)	9 (82)
Intermediate fit	53 (30)	14 (33)	19 (31)	19 (31)	1 (9)
Frail	45 (26)	9 (21)	21 (34)	14 (23)	1 (9)
**ECOG**					
0	90 (51)	17 (40)	33 (54)	33 (54)	7 (64)
1	68 (39)	22 (52)	20 (33)	22 (36)	4 (36)
2	17 (10)	3 (7)	8 (13)	6 (10)	

Data are reported as number (percentage), except for the IQR range.

Abbreviations. Id, ixazomib-dexamethasone; ICd, ixazomib-cyclophosphamide-dexamethasone; ITd, ixazomib-thalidomide-dexamethasone; IBd, ixazomib-bendamustine-dexamethasone; IQR, interquartile range; ISS, International Staging System stage; LDH, lactate dehydrogenase; ULN, upper limit of normal; FISH, fluorescence *in situ* hybridization; R-ISS, Revised ISS; ECOG, Eastern Cooperative Oncology Group performance status.

Table S4. Grade 1-2 and grade 3-4 adverse events occurred during the induction phase

	Id		ICd		ITd		IBd	
	**G 1-2 N=41**	**G 3-4 N=41**	**G 1-2 N=59**	**G 3-4 N=59**	**G 1-2 N=60**	**G 3-4 N=60**	**G 1-2 N=11**	**G 3-4 N=11**
**Hematologic**	1 (2)	2 (5)	3 (5)	7 (12)	5 (8)	5 (8)	2 (18)	2 (18)
Anemia	1 (2)	1 (2)	2 (3)	2 (3)	2 (3)	1 (2)	2 (18)	
Leukopenia						1 (2)		
Neutropenia		2 (5)	1 (2)	4 (7)		3 (5)		2 (18)
Thrombocytopenia			1 (2)	2 (3)	3 (5)	1 (2)		
**Non-hematologic**	28 (68)	7 (17)	33 (56)	11 (19)	43 (72)	29 (48)	5 (45)	4 (36)
**Dermatological**	5 (12)	1 (2)	4 (7)	1 (2)	8 (13)	8 (13)	3 (27)	
**Gastrointestinal**	11 (27)	1 (2)	18 (31)	3 (5)	20 (33)	5 (8)	2 (18)	1 (9)
Diarrhea	3 (7)		7 (12)	3 (5)	4 (7)	2 (3)	1 (9)	
Nausea/vomiting	8 (20)	1 (2)	8 (14)	1 (2)	7 (12)		1 (9)	1 (9)
**General**	12 (29)	1 (2)	10 (17)		13 (22)	4 (7)	2 (18)	1 (9)
Fatigue	6 (15)		4 (7)		8 (13)			
**Infection**	5 (12)	1 (2)	8 (14)	3 (5)	8 (13)	5 (8)	2 (18)	
**Nervous**	11 (27)	3 (7)	14 (24)	4 (7)	26 (43)	10 (17)	1 (9)	1 (9)
Peripheral neuropathy	3 (7)	2 (5)	12 (20)	1 (2)	18 (30)	4 (7)	1 (9)	
**Renal**			2 (3)	2 (3)	1 (2)	1 (2)		
**Vascular**	2 (5)	1 (2)	2 (3)	2 (3)	4 (7)	5 (8)		1 (9)
Thromboembolism					2 (3)	2 (3)		

Data are presented by treatment arm and reported as number (percentage).
Abbreviations. Id, ixazomib-dexamethasone; ICd, ixazomib-cyclophosphamide-dexamethasone; ITd, ixazomib-thalidomide-dexamethasone; IBd, ixazomib-bendamustine-dexamethasone; G, grade.

Table S5. Grade 1-2 and grade 3-4 adverse events occurred during the ixazomib maintenance phase

	**G 1-2 N=102**	**G 3-4 N=102**
**Hematologic**	1 (1)	2 (2)
Anemia	1 (1)	
Thrombocytopenia		2 (2)
**Non-hematologic**	39 (38)	14 (14)
**Dermatological**	13 (13)	4 (4)
**Gastrointestinal**	17 (17)	2 (2)
Diarrhea	5 (5)	1 (1)
Nausea/vomiting	15 (15)	1 (1)
**General**	20 (20)	4 (4)
Fatigue	5 (5)	1 (1)
**Infection**	1 (1)	2 (2)
**Nervous**	19 (19)	
Peripheral neuropathy	16 (16)	
**Renal**	1 (1)	1 (1)
**Vascular**	2 (2)	1 (1)
Thromboembolism		1 (1)

Data are reported as number (percentage).
Abbreviations. G, grade.

Supplementary figures

Figure S1. Study flow

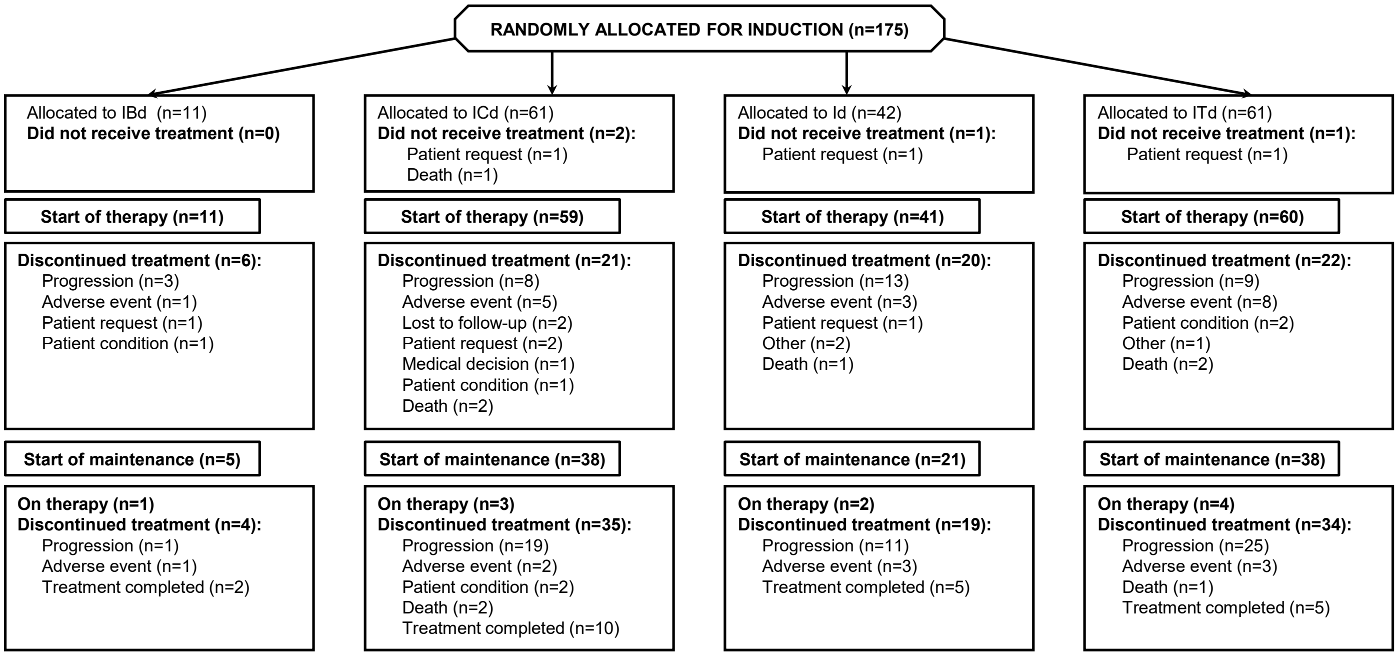

Abbreviations. Id, ixazomib-dexamethasone; ICd, ixazomib-cyclophosphamide-dexamethasone; ITd, ixazomib-thalidomide-dexamethasone; IBd, ixazomib-bendamustine-dexamethasone; n, number.

Figure S2. Kaplan–Meier curve for progression-free survival (PFS) from the start of maintenance in patients (n=102) who completed the induction phase and received ixazomib maintenance

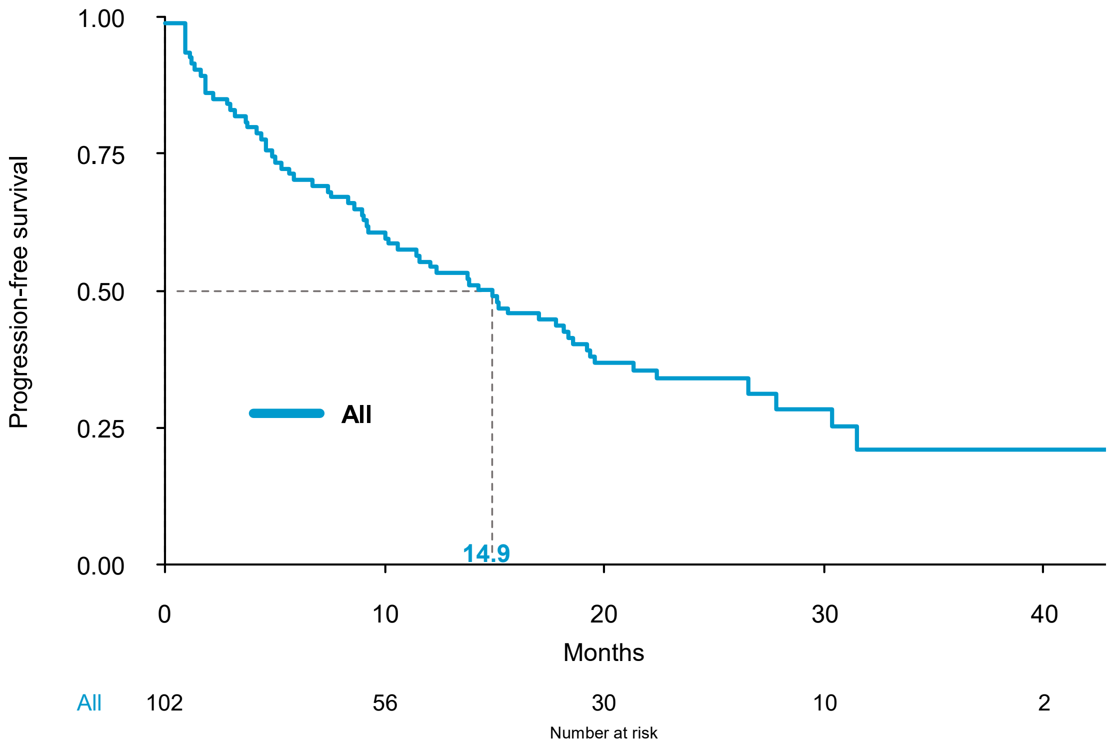

Figure S3. Response improvement from the induction to the maintenance phase

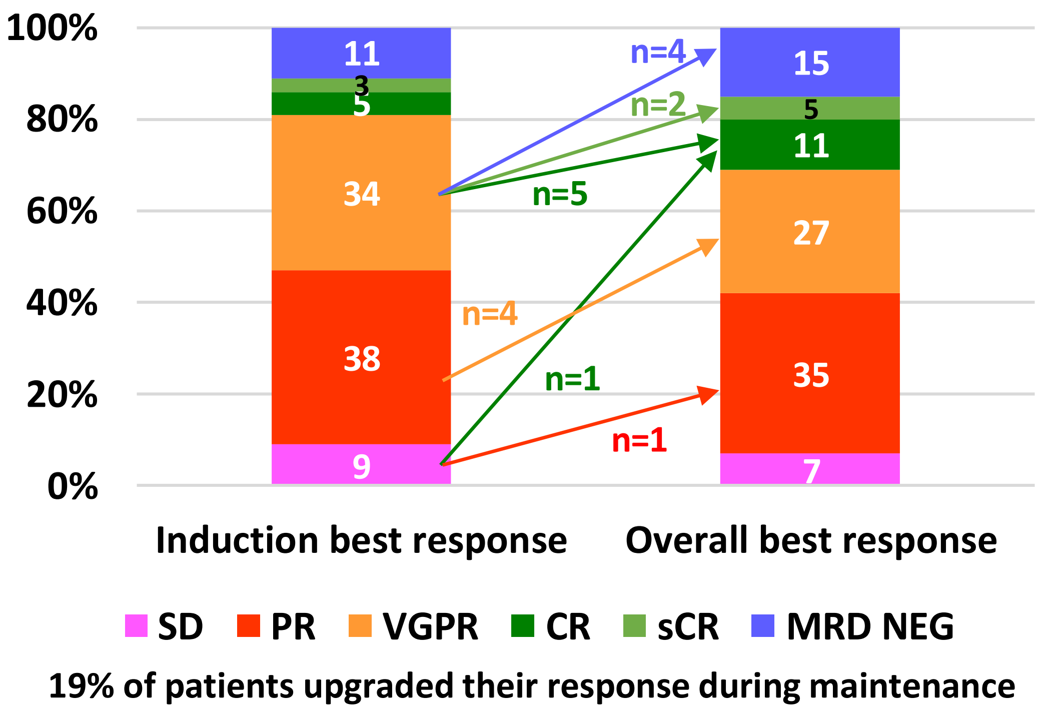

SD, PR, VGPR, CR, sCR, and MRD negativity are defined according to the International Myeloma Working Group consensus criteria for response and minimal residual disease assessment in multiple myeloma.[5]

Abbreviations. SD, stable disease; PR, partial response; VGPR, very good partial response; CR, complete response; sCR, stringent complete response; MRD NEG, minimal residual disease negativity; n, number.

References

1 Thall PF, Simon RM, Estey EH. Bayesian sequential monitoring designs for single-arm clinical trials with multiple outcomes. *Stat Med* 1995; **14**: 357–379.

2 Thall PF, Simon RM, Estey EH. New statistical strategy for monitoring safety and efficacy in single-arm clinical trials. *J Clin Oncol* 1996; **14**: 296–303.

3 Thall PF, Sung H. Some extensions and applications of a Bayesian strategy for monitoring multiple outcomes in clinical trials. *Stat Med* 1998; **17**: 1563–80.

4 U.S. Department of Health and Human Services - National Institutes of Health - National Cancer Institute. Common Terminology Criteria for Adverse Events (CTCAE). Version 4.03, June 14, 2010. 2010.https://evs.nci.nih.gov/ftp1/CTCAE/CTCAE_4.03/CTCAE_4.03_2010-06-14_QuickReference_8.5x11.pdf (accessed 13 May2021).

5 Kumar S, Paiva B, Anderson KC, Durie B, Landgren O, Moreau P *et al.* International Myeloma Working Group consensus criteria for response and minimal residual disease assessment in multiple myeloma. *Lancet Oncol* 2016; **17**: e328–e346.
